# *TAF1*, associated with intellectual disability in humans, is essential for embryogenesis and regulates neurodevelopmental processes in zebrafish

**DOI:** 10.1038/s41598-019-46632-8

**Published:** 2019-07-24

**Authors:** Sanna Gudmundsson, Maria Wilbe, Beata Filipek-Górniok, Anna-Maja Molin, Sara Ekvall, Josefin Johansson, Amin Allalou, Hans Gylje, Vera M. Kalscheuer, Johan Ledin, Göran Annerén, Marie-Louise Bondeson

**Affiliations:** 1 Department of Immunology, Genetics and Pathology, Uppsala University, Science for Life Laboratory, Uppsala, 751 08 Sweden; 2 Department of Organismal Biology, Genome Engineering Zebrafish, Science for Life Laboratory, Uppsala University, Uppsala, 752 36 Sweden; 3 Department of Information Technology, Uppsala University, Sweden and Science for Life Laboratory, Uppsala, 751 05 Sweden; 4 Department of Paediatrics, Central Hospital, Västerås, 721 89 Sweden; 5 Research Group Development and Disease, Max Planck Institute for Molecular Genetics, Berlin, 141 95 Germany

**Keywords:** Embryogenesis, Gene expression, RNA sequencing, Genetics research, Neurodevelopmental disorders

## Abstract

The TATA-box binding protein associated factor 1 (TAF1) protein is a key unit of the transcription factor II D complex that serves a vital function during transcription initiation. Variants of *TAF1* have been associated with neurodevelopmental disorders, but *TAF1*’s molecular functions remain elusive. In this study, we present a five-generation family affected with X-linked intellectual disability that co-segregated with a *TAF1* c.3568C>T, p.(Arg1190Cys) variant. All affected males presented with intellectual disability and dysmorphic features, while heterozygous females were asymptomatic and had completely skewed X-chromosome inactivation. We investigated the role of *TAF1* and its association to neurodevelopment by creating the first complete knockout model of the *TAF1* orthologue in zebrafish. A crucial function of human *TAF1* during embryogenesis can be inferred from the model, demonstrating that intact *taf1* is essential for embryonic development. Transcriptome analysis of *taf1* zebrafish knockout revealed enrichment for genes associated with neurodevelopmental processes. In conclusion, we propose that functional *TAF1* is essential for embryonic development and specifically neurodevelopmental processes.

## Introduction

Transcription factor II D (TFIID) consists of the TATA-box binding protein (TBP) and 12–14 TBP-associated factors (TAFs), and is part of the preinitiation complex that initiates transcription of RNA polymerase II transcription dependent genes^1–3^. Variants in several TFIID components, TBP^4^, TAF2^5^, TAF6^6^ and TAF13^7^, have been associated with neurodevelopmental disorders, proposing a fundamental function of TFIID during embryonic development and especially neurodevelopment. TAF1 is the largest TAF unit of the TFIID complex and plays a key role in the preinitiation complex by facilitating binding to promoter regions^2^. The *TAF1* gene (GRCh37/hg19, chrX:70586114–70685855, NM_004606.4) includes 39 exons and encodes more than 20 coding and non-coding transcripts expressed in various tissues, including the central nervous system^8^.

Coding germline variants of *TAF1* have been reported to cause intellectual disability (ID). Two *TAF1* missense variants were identified in two families within a cohort study including 405 unresolved families with X-linked ID (XLID)^9^, in which two individuals from the family reported here were included (clinical data was not presented). The cohort study was followed by a comprehensive report by O’Rawe *et al*. of 14 males with syndromic XLID (MIM: 300966) from eleven unrelated families who presented with nine different single nucleotide variants (eight missense variants and one splice site variant) and two duplications including *TAF1*. They also demonstrated that asymptomatic female carriers with pathogenic missense variants in *TAF1* from two independent families displayed skewed X-chromosome inactivation (XCI)^10^, which was recently confirmed by Hurst *et al*. in a third case, a mother carrying a TAF1 p.Ser1600Gly substitution that caused XLID in her son^11^. A non-coding 2.6 kb insertion of a SINE-VNTR-*Alu* (SVA)-type retrotransposon in intron 32^8^ of *TAF1* causes the neurological disorder X-linked dystonia-parkinsonism (XDP; MIM: 313650). XDP is a progressive neurodegenerative disorder characterized by involuntary movements (dystonia), most often developing in adult life in combination with parkinsonism^12–14^. Previous mapping^9^ of protein networks have demonstrated TAF1’s association with the RNA polymerase II complex together with proteins associated with ID, such as ATN1^15^, MLL2^16^, RBM10^17^, USP27X^9^, TBP^4^, TAF2^5^, TAF6^6^ and TAF13^7^. A central role of *TAF1* during embryonic development has been proposed after encountering elevated *Taf1* expression during early fetal development in mice^18^. However, to our knowledge, no extensive functional assessment of *TAF1*’s role during embryonic development has been made and the molecular context of *TAF1*’s association to disease remains elusive.

Here, we report clinical and genetic findings of a large family with a likely pathogenic variant in *TAF1* and demonstrate *TAF1*’s association to neurogenesis by creating the first complete *taf1* knockout model. All affected patients were males who had syndromic XLID with phenotypes overlapping with previously published cases, and asymptomatic female carriers revealed completely skewed XCI as described before^10,11^. The *taf1* zebrafish knockout model showed lethality during embryonic development, establishing a crucial function of *taf1* during embryogenesis. Moreover, transcriptome analysis of *taf1* mutants three days post fertilization (dpf) showed enrichment for genes associated with neurodevelopmental processes, providing the first assessments of *taf1* function during embryonic development.

## Results

### Clinical report

**Figure 1:**
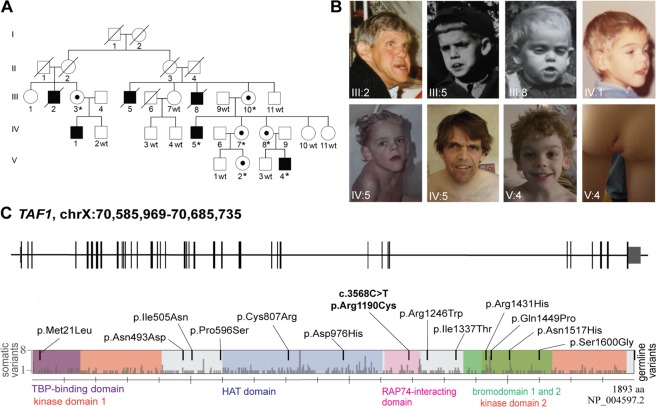
The five-generation family affected with syndromic X-linked intellectual disability (XLID) and the architecture of the *TAF1* gene with missense variants associated with intellectual disability. **(A)** Six males, in the same five-generation family, were initially diagnosed with syndromic intellectual disability. Seventeen family members were genotyped (wt = wild-type, *=variant carrier). **(B)** Photographs show clinical features of the six affected males who presented with a long face, pointed chin, prominent forehead, long philtrum, prominent supraorbital ridge, deep-set eyes, and low-set, protruding, large ears. **(C)** Schematic view of *TAF1* gene and corresponding protein with domains. Germline missense variants associated with intellectual disability are shown above the schematic (black). The likely pathogenic missense variant presented in this report, *TAF1* c.3568C>T, p.(Arg1190Cys), is marked in bold. Light grey bars mark somatic variants reported in the Cosmic cancer database. Domains of TAF1 are kinase domain 1 (amino acid residues 1–414, red) and 2 (residues 1,425–1,872, red), TBP-binding domain (residues 1–140, purple), HAT domain (residues 600–1,009, blue), RAP74-interacting domain (residues 1,110–1,236, pink), and bromodomains 1 and 2 (residues 1,359–1,638, green)^34,59^.

We clinically assessed all affected males from the family (Fig. 1A). They presented with severe (III:2 and III:5), moderate (IV:5 and V:4), or mild (III:8 and IV:1) ID. All affected males had a long face, pointed chin, large hands, prominent forehead, short neck, and low-set, protruding, large ears. A long philtrum, prominent supraorbital ridge, deep-set eyes, and large feet were observed for the majority of males (Table 1). III:2 and III:5 had cataract and died from pneumonia at the ages of 65 and 25 years, respectively. III:8 died from diphtheria at the age of six years. Comprehensive clinical assessments of IV:5 and V:4 revealed additional clinical features, such as oral-pharyngeal dysphagia, a high palate, generalized muscle hypotonia, joint hypermobility, and kyphosis. IV:5 also presented with down-slanted palpebral fissures, scoliosis, and generalized hirsutism. V:4 presented with an asymmetric intergluteal cleft with a sacral dimple (Fig. 1B; Table 1). No hearing impairments or other malformations of the auditory system were noted. Brain magnetic resonance imaging (MRI) of V:4 at two years of age identified no pathological deviations.

**Table 1:** Clinical description of the six male patients and previously reported patients^9–11,32^.

Clinical description	III:2	III:5	III:8	IV:1	IV:5	V:4	Previously reported
Age (years)	65^†^	25^†^	6^†^	54	52	16	
Male	+	+	+	+	+	+	19
Intellectual disability (HP: 0001249)	+	+	+	+	+	+	18
Postnatal growth retardation (HP: 0008897)	−	−	−	−	−	−	11 (2)
Delayed gross motor development (HP: 0002194)	UK	UK	UK	UK	+	+	15
Delayed speech and language development (HP: 0000750)	+	+	+	+	+	+	14
Oral-pharyngeal dysphagia (HP: 0200136)	UK	UK	UK	UK	+	+	9 (1)
Prominent supraorbital ridges (HP: 0000336)	+	+	+	+	+	+	11 (3)
Downslanted palpebral fissures (HP: 0000494)	UK	UK	UK	UK	+	−	10 (3)
Long philtrum (HP: 0000343)	+	+	+	+	+	+	12 (3)
Low-set ears (HP: 0000369)	+	+	+	+	+	+	12 (2)
Protruding ears (HP: 0000411)	+	+	+	+	+	+	11 (3)
Long face (HP: 0000276)	+	+	+	+	+	+	10 (3)
High palate (HP: 0000218)	UK	UK	UK	UK	+	+	10 (2)
Pointed chin (HP: 0000307)	+	+	+	+	+	+	10 (4)
Anteverted nares (HP: 0000463)	−	−	−	−	−	−	10 (4)
Microcephaly (HP: 0000252)	UK	UK	UK	−	−	−	10 (4)
Hypoplasia of the corpus callosum (HP: 0002079)	UK	UK	UK	UK	UK	UK	11 (1)
Generalized hypotonia (HP: 0001290)	UK	UK	UK	UK	+	+	13 (2)
Unusual gluteal crease with sacral caudal remnant and sacral dimple*	UK	UK	UK	UK	−	+	12 (1)
Joint hypermobility (HP: 0001382)	UK	UK	UK	UK	+	+	8 (4)
Autistic behaviors (HP: 0000729)	UK	UK	UK	UK	−	−	10 (3)
Prominent forehead (HP:0011220)	+	+	+	+	+	+	9 (5)
Macrotia (HP:0000400)	+	+	+	+	+	+	1
Broad upturned nose (HP:0000463)	−	−	−	−	−	−	11 (4)
Bulbous nasal tip (HP:0000414)	+	−	−	−	−	−	7 (4)
Short neck (HP:0000470)	+	+	+	+	+	+	0
Deep-set eyes (HP:0000490)	+	+	+	+	−	+	0
Large hands (HP:0001176)	+	+	+	+	+	+	0
Large feet (HP:0001833)	+	+	+	+	+	−	0
Scoliosis (HP:0002650)	UK	UK	UK	UK	+	−	0
Kyphosis (HP:0002808)	UK	UK	UK	UK	+	+	0

The table included features found in the patients of this study and in >9 previously reported patients, (*n*) indicates previous patient reported to not present the phenotype. “+”: Confirmed positive for the clinical characteristic. “−”: Confirmed negative for the clinical characteristic. “UK” (unknown): clinician did not report the status of the phenotype, only features reported by the primary clinician are noted. ^†^Patient is deceased. *Abnormal sacral segmentation [HP: 0008468] and prominent protruding coccyx [HP: 0008472]. An extended table is available (Supplementary Table S1).

### Segregation analysis and bioinformatic predictions propose that the *TAF1* c.3568C>T, p.(Arg1190Cys) variant is likely pathogenic

Linkage analysis of 14 family members using 44 polymorphic microsatellite markers, highlighted a candidate region of 28.3 Mb on Xq11.1–Xq21.32 (Supplementary Data S1). A *TAF1* c.3568C>T, p.(Arg1190Cys) variant was identified within the linked region as part of a cohort X-exome study in which DNA samples from IV:5 and V:4 were included without clinical details^9^. Segregation analysis of 17 family members confirmed the X-linked segregation pattern. Two affected males were hemizygous, five carrier females were heterozygous and ten asymptomatic family members did not carry the *TAF1* variant (Figs. 1A and S1).

The *TAF1* c.3568C>T variant is located in the RAP74-interacting domain (Fig. 1C) and predicted to be disease-causing (MutationTaster^19^), deleterious (SIFT^20^, 0.000) and to affect a conserved base (PhyloP^21^, 3.47 and GERP++^22^, 4.91). Population data in gnomAD reveal an underrepresentation of missense variants in *TAF1* (gnomAD: Z = 5.49, o/e = 0.44,)^23^. Furthermore, no hemizygous or homozygous loss-of-function variants have been reported in SweGen databases^24^. Similarly, gnomAD reports depletion of loss-of-function variants in *TAF1*, (pLI = 1, o/e = 0.00) with only one hemizygous individual harboring an early p.(Ala17Glyfs) loss-of-function variant and one individual hemizygous for a predicted splice acceptor site in the 3′ UTR. In addition, they report one homozygous and 14 hemizygous individuals with an rs200964328 loss-of-function variant; however, this variant only affects a 450 nucleotides short 498 base pair (bp) *TAF1* isoform^23^ and not the canonical protein. In summary, population data demonstrate a strong underrepresentation of missense variants and lack of complete loss-of-function variants in the general population.

### Skewed X-chromosome inactivation in carrier females

**Figure 2:**
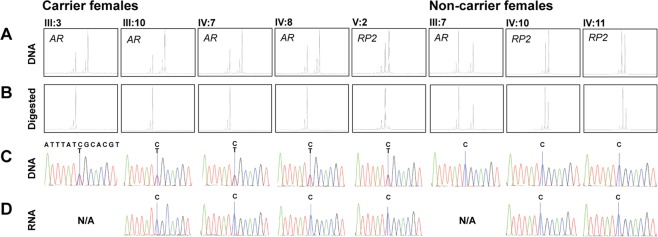
Skewed X-chromosome inactivation in carrier females. **(A)** Fragment length analysis on DNA confirmed biallelic expression of microsatellites (androgen receptor [*AR*] and retinitis pigmentosa 2 [*RP2*]) in all females. **(B)** DNA from carrier females digested with the methylation-sensitive *Hpa*II restriction enzyme showed monoallelic detection, indicating completely skewed X-chromosome inactivation. **(C)** Sanger sequencing of DNA confirmed heterozygosity for the disease-causing variant in carrier females and wild-type sequence for non-carrier females. **(D)** Sequencing of reversed transcribed RNA showed the expression of only wild-type allele in all carrier females and non-carrier females.

The X-chromosome inactivation (XCI) pattern was investigated in five carrier females and three non-carrier females by screening polymorphic microsatellites within the X-linked *RP2* and *AR* genes as previously described^25,26^. The assay revealed a 100% XCI of the mutant allele in all heterozygous carrier females, but normal XCI pattern in the three non-carrier females (Fig. 2A and 2B). Inactivation of the mutant allele was validated by Sanger sequencing of reversed transcribed PCR products derived from carrier females’ RNA, expression of only the wild-type (wt) allele (Fig. 2D).

### *taf1* knockout zebrafish embryos display lethal malformations

**Figure 3:**
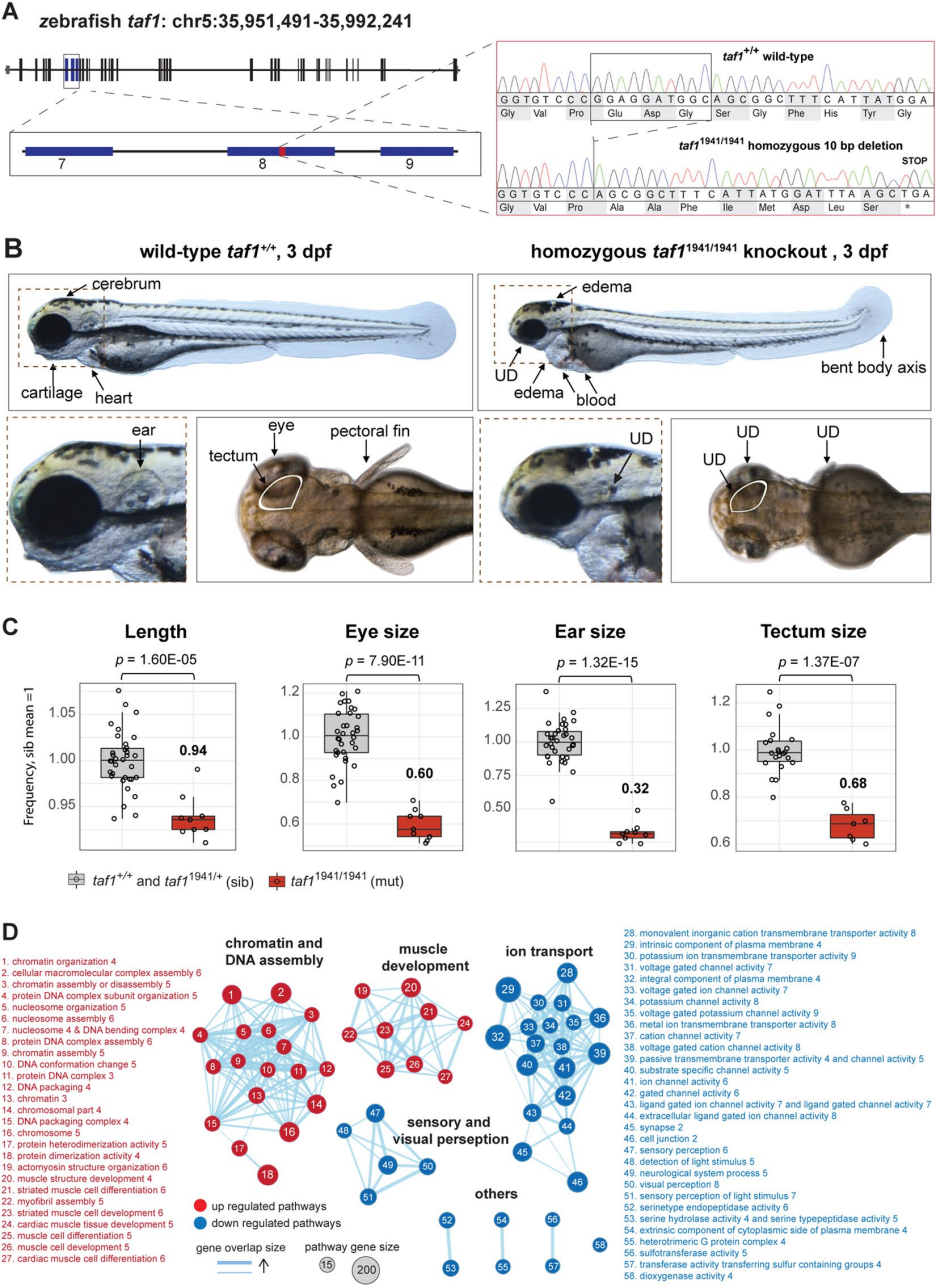
Zebrafish *taf1* knockout model revealed an essential function of *taf1* during embryogenesis and neurodevelopment. **(A)** Sequencing results of *taf1*^uu1941*/*uu1941^ F2 zebrafish embryos confirmed a 10 bp deletion in exon 8, which results in a frameshift and a stop codon, p.Glu362Alafs*9. **(B)** Three days post fertilization (dpf) mutant *taf1*^uu1941/uu1941^ (right) zebrafish embryos showed heart and ventricle edema, blood filled cavities, bent body axis, and general underdevelopment (UD) including short pectoral fins, reduced length and underdeveloped cartilage, eyes, and ears. **(C)** Phenotype quantification demonstrated reduced length, underdevelopment of eyes, ears and tectum when comparing wild-type *taf1*^+/+^ and heterozygous *taf1*^uu1941/+^ (siblings; sib) with *taf1*^uu1941/uu1941^ mutants. Adjusted p-values were generated using Student’s t-test, adjusted with Bonferroni correction. **(D)** Gene set enrichment analysis of all differentially expressed genes revealed four major themes associated with loss of *taf1*. Chromatin and DNA assembly as well as muscle development pathways were upregulated (red) and pathways of ion transport as well as sensory and visual perception pathways were downregulated (blue) in *taf1*^uu1941/uu1941^ zebrafish embryos.

To investigate *taf1*’*s* role during embryogenesis, two mutant lines were successfully bred using Clustered Regulatory Interspaced Short Palindromic Repeats (CRISPR) CRISPR-associated protein 9 (Cas9): *taf1*^uu1931^, with an 8 bp deletion in *taf1* (GRCz10/danRer10, NM_001044785.1) exon 7, resulting in a frameshift of one amino acid followed by a premature stop codon, c.855_862del, p.(Gly296Leufs*2) (Supplementary Fig. S2A), and *taf1*^uu1941^, with a 10 bp deletion in exon 8, resulting in a frameshift of eight amino acids and a premature stop codon, c.1082_1091del, p.Glu362Alafs*9 (Fig. 3A), referred to as mutants or knockouts. The phenotype was assessed in offspring originating from the same clutch of crossing two *taf1*^uu1941/+^ adults and two *taf1*^uu1931/+^ adults. Three dpf, homozygous *taf1* ^uu1941/uu1941^ (*n* = 9) and *taf1* ^uu1931/uu1931^ (*n* = 11) mutants (mut) displayed reduced length, underdeveloped cartilage, eyes and ears, short pectoral fins, heart edema, blood-filled cavities and a dorsally bent body axis compared to siblings (sib; *n* = 34 for both groups; Fig. 3B and Supplementary Fig. S2). The tectum was underdeveloped in *taf1*^uu1941/uu1941^ (*n* = 7) compared to siblings (*n* = 22; Fig. 3C). Measurement of the tectum was obstructed in *taf1*^uu1931/uu1931^, but tectum underdevelopment is likely since similar reduction in head size of about 15% was found in both *taf1*^uu1931/uu1931^ (mut *n* = 9, sib *n* = 33) and *taf1*^uu1941/uu1941^ (*n* = 7, sib *n* = 22; (Supplementary Figs S3Q–T). At 3 dpf, *taf1*^uu1941/uu1941^ (*n = *10) displayed reduced heart beat compared to siblings (*n = *35), but *taf1*^uu1931/uu1931^ did not (mut *n* = 11, sib *n = *33; Supplementary Figs. S3O and S3P). Neither gross gastrointestinal malformations nor musculature malformations were observed. At 5 dpf the phenotype was profound and *taf1*^uu1941/uu1941^ (*n* = 11) displayed severe cranial cartilage defects and a reduced or absent heart beat implied embryonic lethality^27^. Heterozygous embryos (*taf1*^uu1931/+^ and *taf1*^uu1941/+^) displayed no deviations compared to wt (*taf1*^+/+^) zebrafish embryos for any of the observed or quantified phenotypes (Supplementary Fig. S3 and Table S4).

### Transcriptome analysis revealed enrichment of genes important for neurodevelopment processes

Transcriptome sequencing was performed using RNA from pooled mutant (*taf1*^uu1941*/*uu1941^) and sibling (*taf1*^+/+^ and *taf1*^uu1941/+^) zebrafish embryos of the same clutch to investigate *taf1*’s function during embryogenesis. In sample correlation analysis, the three replicates of mutant and sibling embryos were divided into two groups: mutants and siblings (Supplementary Fig. S4). The 10 bp deletion in *taf1*^uu1941*/*uu1941^ mutants was confirmed by Sanger sequencing of cDNA and investigation of transcriptome reads in the Integrative Genomics Viewer^28^. All reads from mutant samples contained the 10 bp deletion. As expected, a few alleles in each of the three sibling pools contained the 10 bp deletion, originating from the heterozygous zebrafish embryos.

A paired differential gene expression analysis reported 6,628 out of 23,919 detected genes as differentially expressed (*padj* <0.01; Supplementary Table S5). PANTHER overrepresentation test^29,30^ on all differentially expressed genes revealed 2.2 enrichment for genes of neuron-neuron synaptic transmission (*padj* <1.3E-04). Out of the 6,628 differentially expressed genes, 612 genes were more than 4-fold downregulated (log_2_ <−2) and 258 genes were more than 4-fold upregulated (log_2_ >2) in mutants. PANTHER overrepresentation test revealed that the 612 most downregulated genes had strongest association to neuromuscular synaptic transmission (7-fold enriched, *padj* < 6.2E-05), specifically by downregulation of GABA receptor genes. The top 258 upregulated genes had strongest association to chromatin assembly (55-fold enriched, *padj* <1.4E-10), specifically by upregulation of histone genes (Supplementary Table S6). In line with PANTHER analysis, an additional gene set enrichment analysis (GSEA; Supplementary Table S5) of all differentially expressed genes, visualized with enrichment map, proposed upregulation of genes associated with chromatin and DNA assembly and muscle development, and downregulation of genes associated with ion transport, and sensory and visual perception (Fig. 3D).

## Discussion

TAF1 is a central protein of the TFIID complex that plays a key role during the initiation of transcription. Several genes encoding proteins of the complex have been associated with ID^4–7^, including *TAF1* that recently was added to the list of about 150 genes linked to XLID^9,31^. In 2015, two missense variants in *TAF1*, p.(Arg1190Cys) and p.(Asn493Asp), were identified in patients with syndromic XLID by investigating 405 families with unresolved XLID^9^. To date, a total of 13 missense variants, one splice site variant and two duplications including *TAF1*^10^ have been reported to cause ID in 19 patients^9–11,32^. However, no functional investigations of *TAF1*’s role during embryogenesis and neurodevelopment have been performed. In this study, we investigated the clinical, genetic, and molecular patterns of a five-generation family with a *TAF1* p.(Arg1190Cys) variant, here determined as likely pathogenic according to ACMG guidelines^33^. Moreover, we also explored TAF1’s function by creating the first *taf1* knockout model that presented a lethal phenotype during embryogenesis and dysregulation of biological processes associated with neurodevelopment.

The male patients investigated in this study presented with XLID, distinct facial features, and additional malformations. In general, the clinical features overlapped with symptoms in previously described patients with *TAF1* missense germline variants. For example, ID, facial dysmorphology and V:4 presented with a peculiar symptom of an asymmetric intergluteal cleft with a sacral dimple, which has been observed in 12 previously reported probands^10^. Further, our report broadens the clinical spectrum of XLID associated with *TAF1* with features such as delayed gross motor development, a prominent forehead, short neck, large hands and feet, and deep-set eyes.

Additional to the clinical comparison with previous cases and segregation analysis, the *TAF1* c.3568C>T, p.(Arg1190Cys) variant was assessed by various bioinformatic prediction tools combined with evaluation of *TAF1*’s susceptibility towards variance using publicly available population databases. GnomAD and SweGen demonstrated a lack of complete loss-of-function variants and underrepresentation of missense variants. The p.(Arg1190Cys) variant is located in the RAP74-interacting domain (amino acid residues 1,110–1,236), where an underrepresentation of germline missense variants in the general population is displayed (14 observed versus 24 expected). The RAP74-interacting domain interacts with TAF7^34^ and supposedly, variants in this domain could interfere with the TAF1–TAF7 interaction, thereby contributing to disease. Further, DECIPHER^35^ reported several affected individuals with *TAF1* variants predicted as pathogenic or likely pathogenic. Collectively, this indicates that some *TAF1* missense variants might be disease-causing and that complete lack of *TAF1* is not compatible with life.

Somatic variants in *TAF1* have been proposed to play a role in tumorigenesis by phosphorylation of p53^36^, which may inhibit overall p53 tumor suppressor activity. In addition, *TAF1* has been proposed as a driver gene for several cancers, such as mammary cancer^37^, colorectal cancer^38^, and clear cell endometrial cancer^39^. The Cosmic database^40^ reports >400 somatic missense variants spread throughout the different domains of the protein (Fig. 1C). Interestingly, the p.(Arg1190Cys) variant (NP_004597.2) investigated in this study has been reported twice in cancer patients as p.(Arg1169Cys) variant (NP_620278.1). Also, one previously reported germline p.(Ile1337Thr) variant (NP_004597.2) causing ID^10^ has been reported in two cancer patients p.(Ile1316Thr), (NP_620278.1). Further, a somatic variant, p.(Arg1225Gln), (NP_620278.1), affecting the same amino acid previously associated with syndromic XLID p.(Arg1246Trp), (NP_ 004597.2), has been reported in two cancer patients. It is well established that somatically mutated genes associated with cancer can be associated with ID when mutated in the germline^41–43^.

The pathogenicity of the *TAF1* p.(Arg1190Cys) variant was further strengthened by the observed 100% skewed XCI in five carrier females, and normal XCI in three non-carrier females of the same family. Skewed XCI in females carrying pathogenic XLID variants is a recognized phenomenon^44^ and has been reported in females heterozygous for pathogenic *TAF1* variants in three independent families before^10,11^. The linkage analysis of the family presented here revealed recombination of the maternal X-chromosome in female IV:11. The recombination resulted in carriership of 65%–79% of the affected X-chromosome and 21%–35% of the wt X-chromosome (Supplementary Fig. S5). The 21%–35% of the wt X-chromosome included *TAF1* and IV:11 was thus found not to be a carrier of the disease-causing variant. In accordance with the other two non-carrier females, IV:11 did not show skewed XCI. The fact that IV:11 carried 65%–79% of the disease-causing X-chromosome but not the disease-causing variant, strengthens the hypothesis that the skewed XCI is caused by the p.(Arg1190Cys) variant. Generally, skewed XCI is thought to arise because of selective advantage of wt cells or disadvantage of mutant cells^45^. However, the mechanism leading to skewed XCI in females carrying pathogenic *TAF1* variants is unclear and, even though it is not within the scope of this article, further studies are needed to clarify the impact of *TAF1* variants on cell survival.

Full knockout models of *TAF1* orthologues to investigate *TAF1* function have so far been missing. However, a study in mice reported that *Taf1* expression is drastically elevated during embryonic development and is then decreased and maintained at stable levels from post-natal week 3 and onwards^18^, proposing an essential function of *Taf1* during embryogenesis. The absence of hemi- and homozygous loss-of-function variants in the protein coding part of the canonical *TAF1* isoform in human population databases proposes that the complete loss of *TAF1* is lethal. To further illuminate *taf1* function during embryonic development we established the first adequate *taf1* knockout model by creating two CRISPR/Cas9 edited zebrafish lines. The reduced *taf1* RNA expression suggested that the frameshift variants, resulting in premature stop codons, led to nonsense-mediated decay of mutant RNA. Both lines presented with a similar phenotype and demonstrated that germline loss of *taf1* resulted in general developmental delay and was embryonically lethal. The utilization of two independent single guide RNAs (sgRNAs) excluded off-target effects as the cause for the lethal phenotype. The minor phenotypical differences between *taf1*^uu1931/uu1931^ and *taf1*^uu1941/uu1941^ could be explained by the differential functionality of the truncated protein, line-specific genetic variation or minor guide-specific off-target effects. Quantification of tectum size was performed as a proxy for brain malformations previously reported in patients with *TAF1* variants. At 3 dpf, a reduction of notably 32% was measured in the mutants (*taf1*^uu1941/uu1941^) compared to siblings (*taf1*^uu1941/+^ and *taf1*^+/+^). This is in line with a prior study that assessed the effect of *taf1* knockdown by morpholino, which displayed a 10% decrease in tectum size^10^. The increased reduction observed in our model is likely linked to the advantage of examining animals with complete loss of *taf1*. The absence of malformations in heterozygous zebrafish mutants in our study confirms that monoallelic expression of *taf1* is sufficient for normal embryonic development and suggests why previous attempts did not reveal measurable phenotypic deviations in F0 *taf1* mutants^10^.

To illuminate pathways affected by loss of *taf1*, we investigated the enrichment for genes of certain biological processes in our set of differentially expressed genes. Overrepresentation analysis of all differentially expressed genes (*n* = 6,628) revealed a 2.2 overrepresentation of genes associated with neuron-neuron synaptic transmission. Genes more than 4-fold downregulated *(n* = 612) were 7-fold enriched for neuromuscular synaptic transmission and the genes more than 4-fold upregulated (*n* = 258) were 55-fold enriched for chromatin assembly, proposing that *taf1* regulates genes important for neurodevelopmental processes (Supplementary Table S6). More specifically, histone genes were particularly upregulated and GABA receptor genes were particularly downregulated in *taf1* mutant zebrafish embryos. Variants in chromatin remodeling genes^46,47^, and histone genes in particular^48,49^, as well as GABA genes^50^ have repeatedly been linked to ID. In line with the PANTHER results, GSEA result of all differentially expressed genes, visualized using enrichment map, highlighted chromatin and DNA assembly pathways as well as ion transport pathways as two major themes. A recent study by Aneichyk *et al*. established XDP’s association with an intronic variant of *TAF1*^8^ causing reduced *TAF1* expression in stem cell-derived neuronal cells and, likely, XDP^12–14^. Transcriptomics on XDP patient-derived neuronal cells implied disturbed regulation of neuronal processes and axon guidance by analysis on a subset of differentially expressed genes. Hurst *et al*. recently investigated expression of 86 neuronal ion-channel genes in *TAF1* deficient neuronal cells of which eight genes showed differential expression^11^. Four of these genes orthologs (*ccnd1, cacna1g, kcnj14* and *asic2*) were also differently expressed in zebrafish mutants. Additionally, ATP-sensitive inward rectifier potassium channel 12 (*kcnj12*), belonging to the same gene family as *kcnj14*, was the most underexpressed gene in our data (log_2_ −6.1). Data provided by Aneichyk *et al*. displayed a moderate upregulation of *kcnj12*. Hurst *et al*. highlighted reduced expression of Cyclin D1 (*CCND1)* that is associated with proliferation and is important for cerebellar development and highlighted as dysregulated in patients with *TAF1* variants by Ballouz *et al*. (https://www.bio-rxiv.org/content/10.1101/128439v2). We observed a slightly reduced expression of *ccnd1* in our dataset (log_2_ −0.49). Apart from *ccnd1*, our dataset revealed striking upregulation of *ccna1* (log_2_ 3.6) and to a lesser extent dysregulation of other cyclins, including *a2, b1, b3, c, e2, g1, l1* and *t2*. Up to this point, no key pathways or genes have been highlighted from the combined results of previous RNA studies. Compared to Aneichyk and Hurst *et al*., our analysis has the advantage of investigating all differentially expressed genes of a germline knockout during embryonic development. The result highlights chromatin processes and ion transport processes as particularly dysregulated, both processes have previously been linked to neurodevelopment. Combined, these results advocate that germline variants in *TAF1* could cause XLID by dysregulation of genes essential for neurodevelopment. However, even though it is not within the scope of this study, further assessments are needed to clarify *TAF1*’s relation to synaptic transmission, chromatin assembly and ion transport in humans to better understand the mechanism causing syndromic XLID.

In conclusion, we present a five-generation family with six males affected by syndromic XLID associated with *TAF1* c.3568C>T, p.(Arg1190Cys) hemizygosity. Five carrier females were asymptomatic and showed 100% skewed XCI. We investigated the role of *taf1* during embryonic development by creating the first germline *taf1* knockout model, where the lethal outcome in zebrafish embryos illuminated the importance of functional *taf1* during embryonic development. Subsequent transcriptome analysis highlighted several genes important for neuronal processes to be associated with *taf1* function, proposing that human TAF1 may be a key regulator of neurogenesis. Further investigation of *TAF1* missense variants’ association to disturbed neurodevelopmental processes are important as they may help to understand the full etiology of syndromic XLID associated with *TAF1*.

## Materials and Methods

### Ethical consent

Prior to the initiation of the study, the local ethics committee for human research in Uppsala, Sweden, approved this study (Dnr 2012/321). Informed consent for the study was obtained from the participants and legal guardians of the participants below 18 years of age. Consent to publish identifying images in an online, open access journal has been obtained from participants and legal guardians of participants below 18 years of age. All clinical investigations and genetic analyzes were conducted in accordance with the guidelines of the Declaration of Helsinki. All animal experimental procedures were approved by the local ethics committee for animal research in Uppsala, Sweden (permit number C161/4).

### Clinical assessment

The five-generation family consisted of 32 members with 6 out of 20 males diagnosed with a syndromic form of XLID. Comprehensive and continuous clinical examinations were performed since early childhood for patient V:4 and since young adulthood for patient IV:5. Patients III:2, III:5, III:8, and IV:1 were clinically assessed based on photographs and family history. Phenotype data has been submitted to ClinVar.

### Polymerase chain reaction and Sanger sequencing

For genetic analysis, peripheral blood samples were collected from 17 family members. DNA and RNA was extracted according to standard protocols.

Polymerase chain reaction (PCR) and Sanger sequencing were performed on genomic DNA and reverse-transcribed cDNA. Approximately 500 ng of RNA was used for cDNA synthesis with oligo(dT) according to Maxima H Minus First Strand cDNA Synthesis Kit (Thermo Fisher Scientific, Waltham, MA). *Taq* PCR was performed on 1 μl of cDNA or 50 ng of DNA at 95 °C for 5 min, 20 cycles (95 °C 20 sec, 65–55 °C 30 sec, 72 °C 1 min) and 25 cycles (95 °C 20 sec, 55 °C 30 sec, 72 °C 1 min) following a standard protocol (Applied Biosystems, Waltham, MA). Sanger sequencing was performed on a 3130XL ABI Genetic Analyzer using the ABI PRISM BigDye Primer v3.0 Cycle Sequencing Ready Reaction Kit (Applied Biosystems, Waltham, MA). Primer sequences are stated in Supplementary Table S2. Variant data has been submitted to ClinVar.

### Assays for X-chromosome inactivation assessment

One μg of genomic DNA isolated from peripheral blood was digested with FastDigest *Hpa*II in a 20 µl reaction volume using 1 µl of restriction enzyme according to standard protocol (Thermo Fisher Scientific, Waltham, MA). Amplification of the androgen receptor (*AR*) microsatellites was carried out in a 20 µl reaction containing 0.1 mM of dNTP, 250 nM of each primer, 1.25 mM of MgCl_2_, 2 µl of buffer, 1 U of *Taq* polymerase, and 50 ng of DNA or 2 µl of digested DNA, with primers and cycling conditions as previously described^25^. Amplification of the retinitis pigmentosa 2 (*RP2*) gene microsatellites was performed as previously described^26^ with an input of 50 ng of DNA or 2 µl of digestion product. 5′ primers were marked with (6)-Carboxyfluorescein protein (/56-FAM; Supplementary Table S2). Genotyping was performed through a fragment length analysis (FLA), as described in the Supplemental Data 1, and analyzed with the GeneMapper™ software (Thermo Fisher Scientific, Waltham, MA). Primer sequences are stated in Supplementary Table S2.

### CRISPR/Cas9 target design

Two sgRNAs targeting the single zebrafish *taf1* gene with no predicted off-target effects were designed using the online software CHOPCHOP^51^: one targeting exon 7 (5′-GGG CTA AGA AAA AAT CAG GGT GG-3′) and the other targeting exon 8 (5′-GGT GTC CCG GAG GAT GGC AGC GG-3′). The sgRNAs were prepared as previously described^52^, creating a fragment consisting of the T7 promotor, the targeted gene specific sequence, and the guide core sequence. The sgRNAs were synthesized by *in vitro* transcription using the HiScribe T7 High Yield RNA Synthesis Kit (New England Biolabs, Ipswich, MA). *Cas9* mRNA was prepared by *in vitro* transcription with the mMESSAGE mMACHINE T3 Transcription Kit (Life Technologies, Carlsbad, CA) using 500 ng of linearized plasmid that was retrieved from 5 μg of p-T3TS-nCas9n plasmid (plasmid #46757; Addgene, Cambridge, MA) digested with *Xba*I (New England Biolabs, Ipswich, MA). The products were purified, and their integrity were assessed using a denaturation gel.

### Animals

Fertilized zebrafish (*Danio rerio*) eggs (AB strain) were obtained by natural spawning. Embryos were injected at the one-cell stage with 150 pg of *Cas9* mRNA and 50 pg of each sgRNA in RNase-free H_2_O as previously described^52^ and maintained at 28.5 °C in E3 medium^53^. The efficiency of the targets was estimated by the CRISPR-Somatic Tissue Activity Test (STAT) methodology in eight embryos at two days post-injection, as previously described^54^. The injected founder zebrafish (F0) were raised and incrossed. For genotyping the F1 zebrafish, DNA was extracted from a 1–3 mm amputation of the adult zebrafish caudal fin by lysing the tissue in 30 μl of 50 mM NaOH for 20 min at 95 °C, adding 60 μl of 0.1 mM Tris and diluting the obtained material (1:10). For the initial genotyping step, FLA analysis was used. Two μl of DNA (50–200 ng) was added to Platinum *Taq* DNA Polymerase^55^. The PCR mix was incubated at 94 °C for 12 min followed by 35 cycles (94 °C 30 sec, 57 °C 30 sec, 72 °C 30 sec) and 72 °C for 10 min. Size determination was carried out on a 3130XL ABI Genetic Analyzer (Applied Biosystems, Waltham, MA) and the data was analyzed using the Peak Scanner Software (Thermo Fisher Scientific, Waltham, MA). For the fish that screened positive for the variant, the FLA results were confirmed by Sanger sequencing as described above.

### Phenotype assessment

Two strains with alleles containing frameshift deletions resulting in premature stop codons (*taf1*^uu1931^ and *taf1*^uu1941^) were selected for further experiments. The identified founders were crossed with wt zebrafish (AB strain), and their adult offspring were genotyped. Heterozygous carriers from both mutant lines were individually crossed and the offspring was observed. Phenotypic observation and quantification of eye, ear and length were performed on 43 and 45 embryos from the same clutch of *taf1*^uu1941^ and *taf1*^uu1931^, respectively. Twenty-nine and 42 embryos from the same clutch of *taf1*^uu1941^ and *taf1*^uu1931^ respectively, were raised under normal conditions with the addition of 0.003% 1-phenyl 2-thiourea (PTU) at 6 hours post fertilization to reduce pigmentation, which allowed measurement of head and tectum size as demonstrated before^56^. Eye, ear, head and tectum area as well as length was measured with ImageJ on images generated with a Leica microscope or the VAST bioImager™. Heart rate was measured in 44 and 45 embryos from the same clutch of *taf1*^uu1941^ and *taf1*^uu1931^, respectively by an in-house script quantifying the mean heart beats per minute from a ten second movie generated by Vertebrate Automated Screening Technology (VAST) bioImager™ platform. Twenty-four *taf1*^uu1941^ were kept until 5 dpf to assess the lethal phenotype. Embryos were sacrificed and genotyped by FLA and/or Sanger sequencing as described above (primer sequences are given in Supplementary Table S2). Data was visualized in boxplots generated by ggplot in R environment. Significance was established using Student’s t-test adjusted with Bonferroni correction. Specific genotype distributions of clutches are available in Supplementary Table S3.

### RNA extraction from zebrafish embryos

Zebrafish embryos (3 dpf) from crossing three pairs of *taf1*^uu1941/+^ heterozygous adults were sedated, washed in PBS, sorted based on their phenotype (normal or mutant), and collected in 10 μl RNAlater /embryo (Ambion, Thermo Fisher Scientific, Waltham, MA). Mutant (*taf1*^uu1941/uu1941^) and sibling (*taf1*^+/+^ and *taf1*^uu1941/+^) zebrafish embryos were pooled as follows: *n* = 10 for pair 1 and *n = *20 for pairs 2 and 3. Samples were held on ice and then incubated at 4 °C for 24 hours. The embryos were stored at −20 °C until the day of extraction. For RNA extraction, samples were thawed at room temperature and RNAlater was removed by pipetting. Samples were homogenized and lysed in 0.5 ml of TRIzol according to the manufacturer’s protocol (Ambion, Waltham, MA) using a BioVortexer mixer. RNA cleaning was carried out using a Qiagen RNeasy Micro Kit according to the manufacturer’s protocol (Appendix C). RNA quality was assessed using the Agilent 2100 Bioanalyzer and RNA 6000 Nano Kit (Agilent Technologies, Santa Clara, CA).

### RNA library preparation

Libraries were prepared using 190 ng of poly(A)-selected RNA with the TruSeq Stranded mRNA sample preparation kit according to the manufacturer’s protocol (Illumina Inc., San Diego, CA). The quality of the libraries was evaluated using the TapeStation (D1000 ScreenTape, Agilent Technologies), and the adapter-ligated fragments were quantified by qPCR using the Library Quantification Kits from Illumina (KAPA Biosystems, Boston, MA) on a StepOnePlus instrument (Applied Biosystems, Waltham, MA) prior to cluster generation and sequencing.

### Transcriptome sequencing and analysis

RNA sequencing was carried out on Illumina’s HiSeq2500 with a PE125 read length (HCS v2.2.58/RTA v1.18.64) according to the manufacturer’s instructions (Illumina, San Diego, CA). Demultiplexing and conversion to FASTQ format was performed using the bcl2fastq2 (v2.19.1.403) software, provided by Illumina. Additional statistics on sequencing quality were compiled with an in-house script from the FASTQ, RTA, and bcl2fastq2 output files.

RNA sequencing data quality was assessed with FastQC (v0.11.5) and the RSeQC script package (v0.1.0). TrimGalore (v0.4.1) was used for trimming adapter contamination, followed by alignment to the reference sequence (GRCz10/danRer10) using Star (v2.5.1). FeatureCounts (v1.5.1) was used to receive transcript counts, and DESeq2 (v1.19.13) was used for paired differential gene expression analysis. An adjusted *p*-value < 0.01, corrected by the Benjamini-Hochberg procedure, was used to select significantly differentially expressed transcripts. PANTHER overrepresentation test (v13.1)^29,30^ corrected for multiple testing using Bonferroni correction was used to investigate enrichment of biological functions. GSEA was performed on all differentially expressed genes (adjusted *p*-value < 0.01) with 1109 zebrafish pathways retrieved from GO2MSIG^57^ filtered for minimum 15 genes, and maximum 200 genes, per pathway. The GSEA result was visualized with enrichment map using yFiles Organic layout with a Q-value < 0.01 and edge cut-off <0.0375, according to protocol^58^.

## Supplementary information

Supplementary information

Table S5

Table S1

## Data Availability

All data is available upon request.
