# Supplementary material for: TAF1, associated with intellectual disability in humans, is essential for embryogenesis and regulates neurodevelopmental processes in zebrafish
Source: Sci Rep. 2019 Jul 24;9:10730. doi: 10.1038/s41598-019-46632-8 (PMC6656882; doi:10.1038/s41598-019-46632-8)
Supplement: Supplementary file 1 — Supplementary information [file 41598_2019_46632_MOESM1_ESM.docx]

***TAF1*, associated with intellectual disability in humans, is essential for embryogenesis and regulates neurodevelopmental processes in zebrafish**

Sanna Gudmundsson^1*^, Maria Wilbe^1^, Beata Filipek-Górniok^2^, Anna-Maja Molin^1^, Sara Ekvall^1^, Josefin Johansson^1^, Amin Allalou^3^, Hans Gylje^4^, Vera M. Kalscheuer^5^, Johan Ledin^2^, Göran Annerén^1*^, Marie-Louise Bondeson^1*^

^1^Department of Immunology, Genetics and Pathology, Uppsala University, Science for Life Laboratory, Uppsala, 751 08, Sweden.

^2^Department of Organismal Biology, Genome Engineering Zebrafish, Science for Life Laboratory, Uppsala University, Uppsala, 752 36, Sweden.

^3^Department of Information Technology, Uppsala University, Sweden and Science for Life Laboratory, Uppsala, 751 05, Sweden.

^4^Department of Paediatrics, Central Hospital, Västerås, 721 89, Sweden.

^5^Research Group Development and Disease, Max Planck Institute for Molecular Genetics, Berlin, 141 95, Germany.

***Correspondence:** [sanna.gudmundsson@igp.uu.se](mailto:sanna.gudmundsson@igp.uu.se) (SG), [goran.anneren@igp.uu.se](mailto:goran.anneren@igp.uu.se) (GA) [marielouise.bondeson@igp.uu.se](mailto:marielouise.bondeson@igp.uu.se) (MLB)

**Supplementary Information**

**Supplementary data**

**Data S1** Linkage analysis

**Linkage analysis methods:** The linkage analysis was performed on 14 family members (III:3, III:7, III:9, III:10, III:11, IV:2, IV:5, IV:7, IV:8, IV:10, IV:11, V:1, V:3, V:4) with 44 polymorphic microsatellite markers amplified by polymerase chain reaction (PCR; detailed protocols and primer sequences are available upon request). The microsatellites were assessed by fragment length analysis (FLA) on the 3130XL ABI Genetic Analyzer with GeneScan 500 ROX Size Standard (Thermo Fisher Scientific, Waltham, MA). The genotype was determined using the Peak Scanner Software v1.0 (Applied Biosystems, Foster City, CA). A haplotype analysis was carried out with Cyrillic software v2.1.3 and manually. A two-point linkage analysis of the markers and the disease locus was conducted by using the MLINK program of the LINKAGE package v5.1. The mutation rate for the disease locus was set to 0 and the disease gene frequency to 0.0001. The inheritance was set to X-linked recessive inheritance with full penetrance, i.e., hemizygous males with the disease allele would be affected with a probability of one and likewise for homozygous females with the disease allele. The allele frequencies for the markers were assumed to be equal. Physical and genetic distances were obtained from the UCSC genome browser.

**Linkage analysis results:** A linkage analysis using 44 polymorphic microsatellite markers highlighted a candidate region on Xq11.1–Xq21.32 of 28.3Mb (Marshfield: 8.12cM, deCode: 14.9cM). Of these, 12 markers gave a positive logarithm (base 10) of odds (LOD) score, of which 4 had a LOD score >2 at θ = 0 (DXS1275: 2.44, DXS1225: 2.14, DXS986: 2.47, and DXS8114: 2.19). Recombination was observed at two positions for the maternally inherited X-chromosome of IV:11. In addition, 65%–79% of the maternally inherited X-chromosome originated from the X-chromosome carrying the pathogenic variant and 21%–35% originated from the wild-type chromosome. IV:11’s carriership of the pathogenic variant could not be determined using linkage analysis as recombination occurred within the linked candidate region (Fig. S5).

**Data S2. Web resources**

Catalogue of Somatic Mutations in Cancer (Cosmic) database: <http://cancer.sanger.ac.uk/cosmic>

Database of genomic variation and phenotype in humans using Ensembl resources (DECIPHER): <https://decipher.sanger.ac.uk/>

Genomic Evolutionary Rate Profiling (GERP): <http://mendel.stanford.edu/SidowLab/downloads/gerp/>

Genome Aggregation Database (GnomAD): <http://gnomad.broadinstitute.org/>

<http://mendel.stanford.edu/SidowLab/downloads/gerp/index.html>

MutationTaster: <http://www.mutationtaster.org/>

Online Mendelian Inheritance in Man (OMIM) database: <https://www.omim.org/>

Protein Analysis Through Evolutionary Relationships (PANTHER) database: <http://www.pantherdb.org/>

SweGen Frequency Browser <https://swegen-exac.nbis.se/>

Sorting Tolerant From Intolerant (SIFT) algorithm <http://sift.jcvi.org/>

University of California Santa Cruz (UCSC) Genome Browser: <https://genome.ucsc.edu/>

GO2MSIG: http://www.go2msig.org/cgi-bin/prebuilt.cgi?taxid=7955**Supplementary figures**


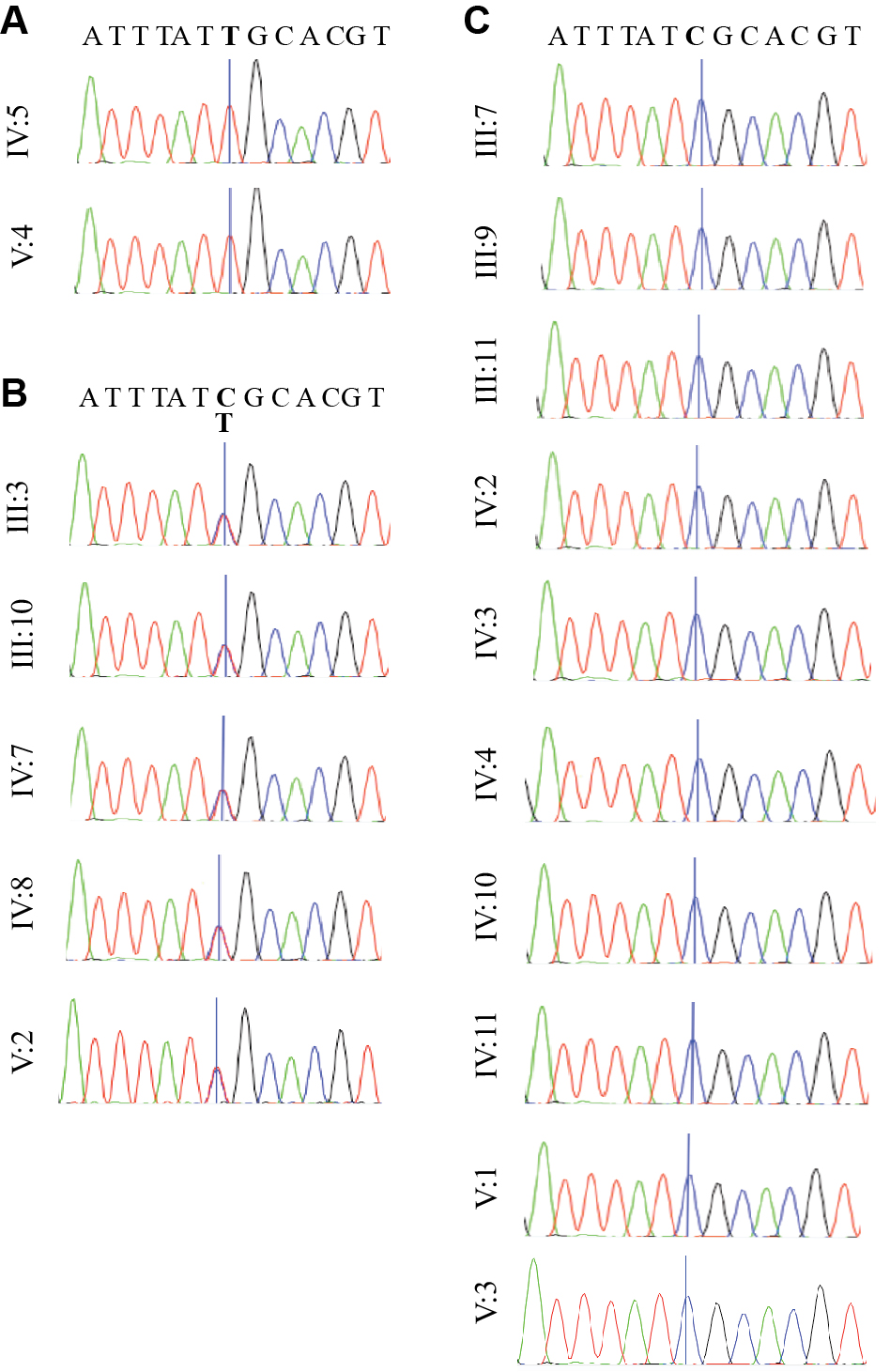


**Figure S1.** *TAF1* c.3568C>T, p.(Arg1190Cys) variant was investigated in 17 family members, including **(A)** two affected hemizygous males, **(B)** five heterozygous carrier females and **(C)** ten non-carrier family members.

**
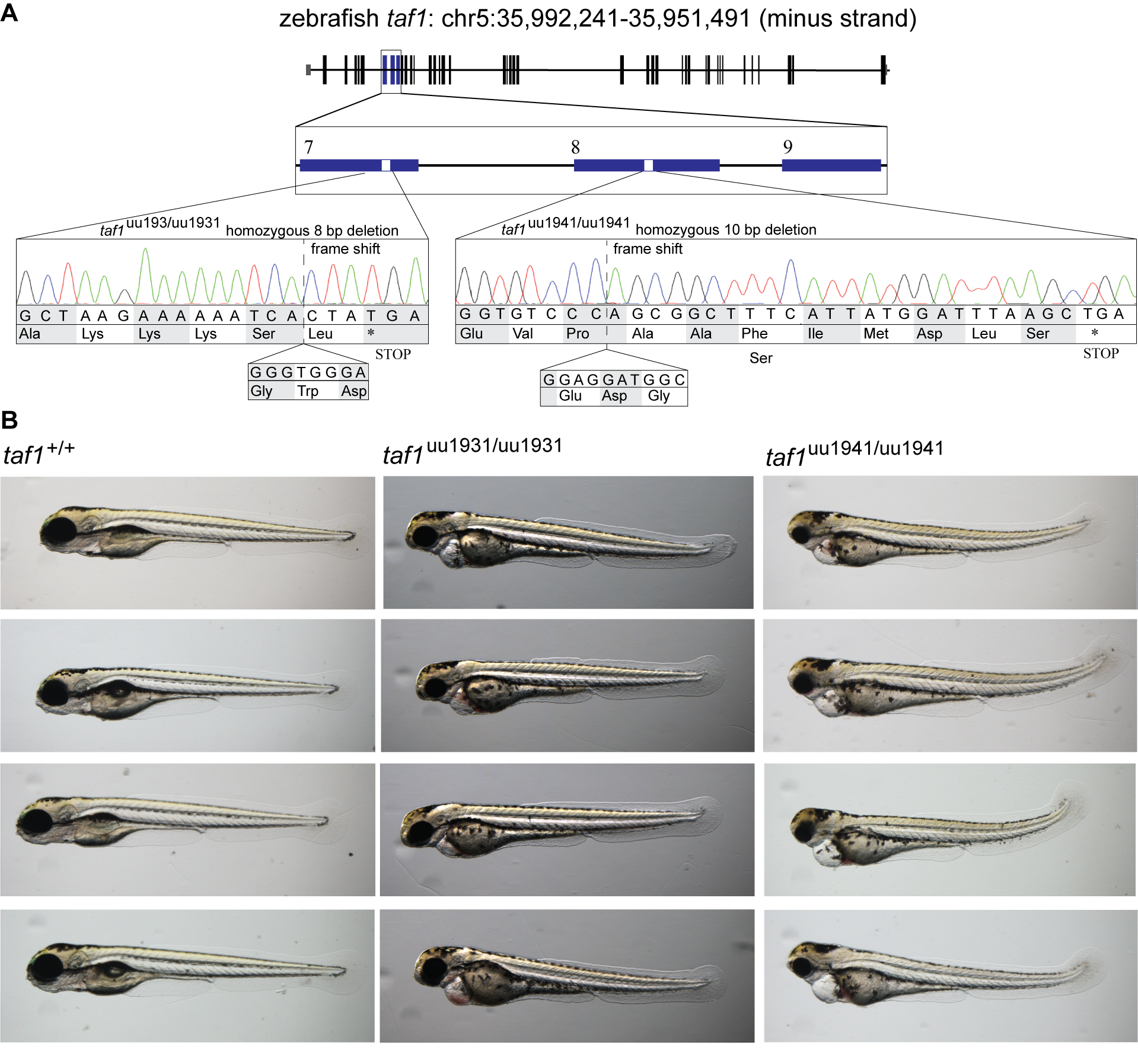
**

**Figure S2.** Comparison of two different *taf1* knockout strains. **(A)** Schematic overview of deletions in two different strains, *taf1*^uu1931/ uu1931^ p.(Gly296Leufs*2) and *taf1*^uu1941/ uu1941^ p.Glu362Alafs*9. **(B)** Four zebrafish embryos of both *taf1*^uu1931/ uu1931^ and *taf1*^uu1941/ uu1941^ at 4 days post-fertilization show a similar abnormal phenotype compared to wild-type *taf1* zebrafish (left). The malformations were lethal and included general developmental delay, reduced pigmentation, underdeveloped craniofacial cartilage, cerebral edema, underdeveloped and malformed ears, dorsally bent body axis, heart edema, blood-filled cavities, short pectoral fin, and underdeveloped eyes with coloboma.


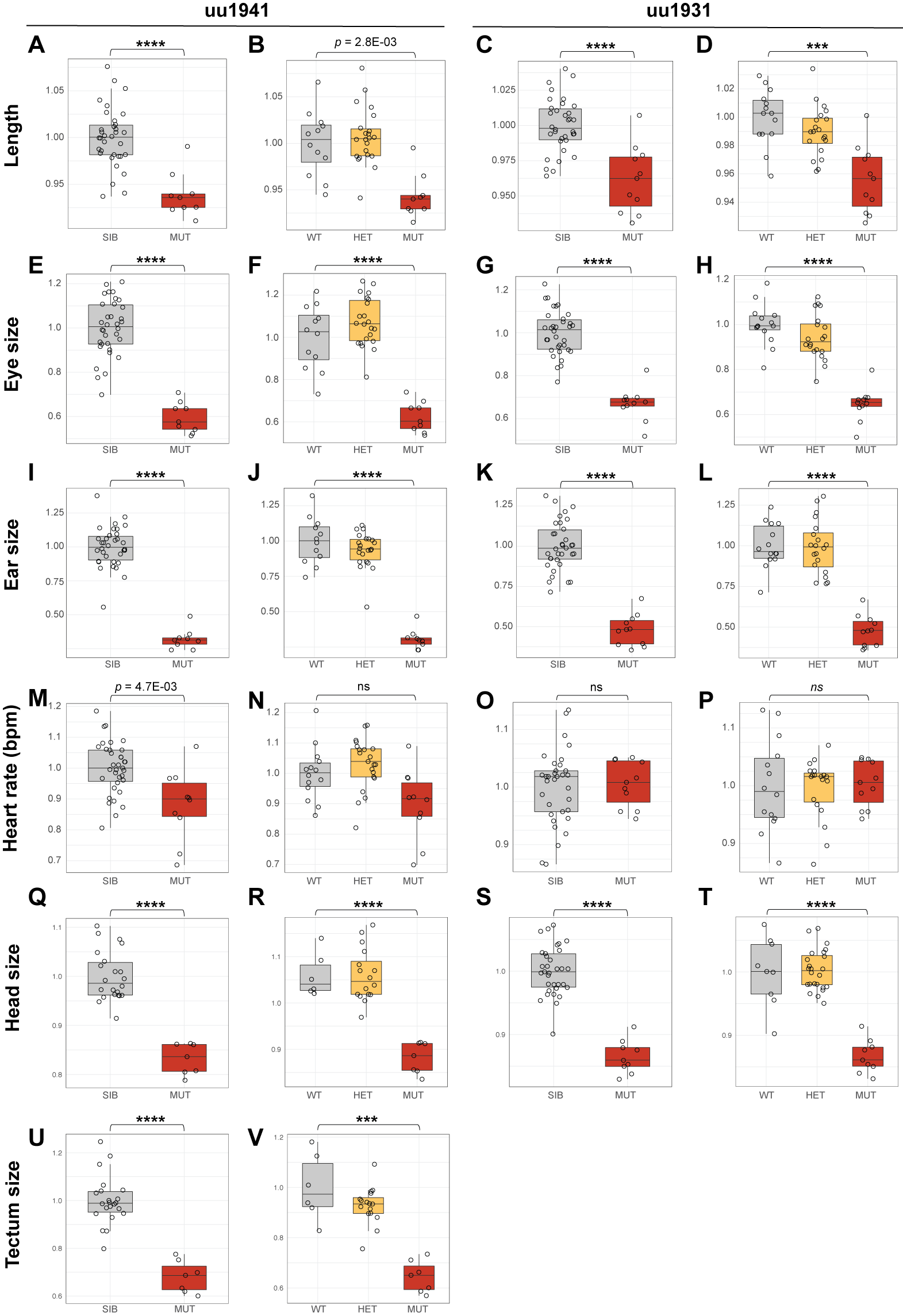


**Figure S3**. Quantification of length, eye size, ear size, heart rate, head size and tectum size in *taf1*^uu1941^ and *taf1*^uu1931^ strains in frequency of wild-type (wt) or siblings (sib) mean of the same clutch. First and third columns display the comparison between sib including wild-type *taf1*^+/+^ and heterozygous *taf1*^+/-^ versus homozygous *taf1*^-/-^ mutants (mut). Second and forth columns display comparison of wt *taf1*^+/+^ versus heterozygous *taf1*^+/-^ (het), and wt versus mut. Homozygous mut showed significant differences in length, eye size, ear size, head size and tectum size in both *taf1*^uu1941^ and *taf1*^uu1931^ strains. Heart rate measured in beats per minute (bpm) was not significant for all groups. No tests displayed a significant difference between wt and het. *** adjusted p-value <0.001 (adjusted by Bonferroni correction), **** <0.0001. Non-significant (ns) adjusted p-values were >0.45, specific p-values (raw and adjusted) are listed in Table S4.


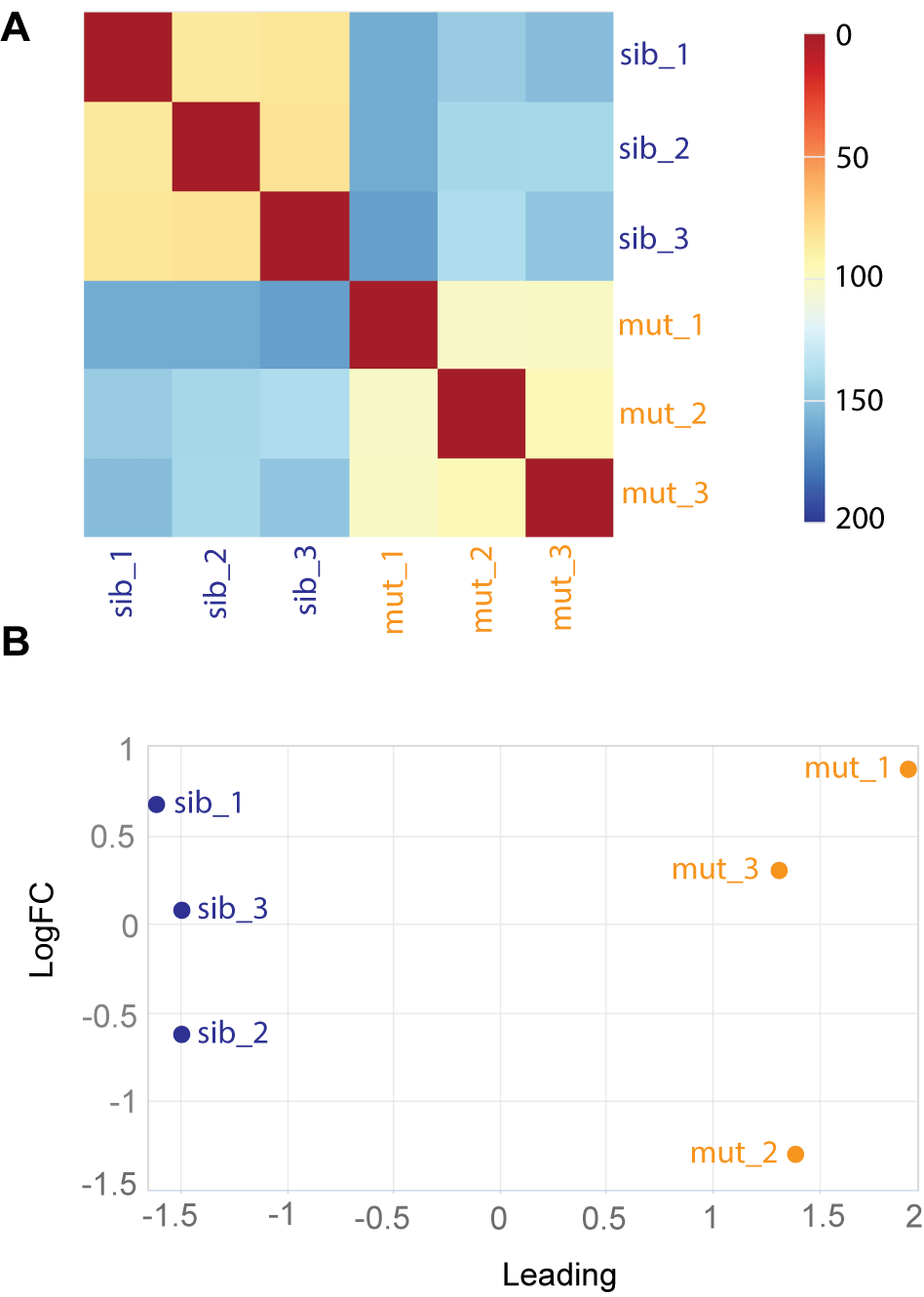


**Figure S4.** Sample correlation was performed for transcriptome data using edgeR and MultiQC. **(A)** A sample distance plot displaying a clear separation into two groups: sibling samples (sib_1, sib_2, and sib_3) and mutant samples (mut_1, mut_2, and mut_3). **(B)** Multidimensional scaling (MDS) plot confirmed the division between wild-type and mutant zebrafish embryo samples and revealed a smaller but detectable difference between the three different clutches, which might be due to environmental factors or specific genetic background within the specific breeding pair.

**
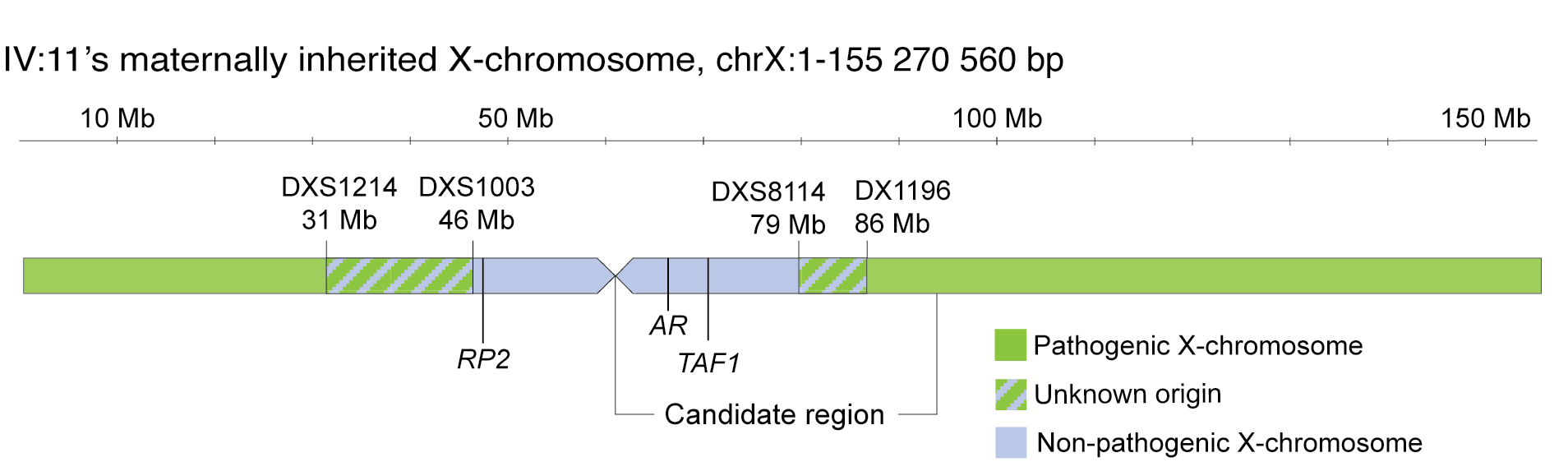
**

**Figure S5.** Schematic overview of the maternally inherited X chromosome of female IV:11 who does not carry the *TAF1* variant. A recombination resulted in an X chromosome constituting both the maternal wild-type X chromosome (blue) and the maternal X chromosome carrying the disease-causing variant (green). The positions of markers at the breakpoints as well as the androgen receptor gene (*AR*) and the retinitis pigmentosa 2 gene (*RP2*) used for studying the X-chromosome inactivation are indicated.

**Supplementary tables**

**Table S1.** Full table of reported phenotypes.

“+”: confirmed positive for the clinical characteristic. “-”: confirmed negative for the clinical characteristic. “UK”: (unknown) clinician did not report the status of the phenotype, only features reported by the primary clinician are noted. “†”: patient is deceased. *abnormal sacral segmentation [HP: 0008468] and prominent protruding coccyx [HP: 0008472]. (*n*) indicates previous patient reported not to present with the phenotype.

*See attached excel file TableS1.*

**Table S2.** Primers used in this study.

|  |  |  |
| --- | --- | --- |
| **Primer Sequence** | **Forward** | **Reversed** |
| Sanger sequencing of variant, patient DNA | AAG GGG AAC GTT TGG AAA TC | GTA TGC GCA CAT AGG CAT CA |
| Sanger sequencing of variant, patient RNA | CTA CTG GCA GCA GGC TCA G | GTT GGA AGG TGG CGC ATT |
| Skewed XCI, androgen receptor gene microsatellites | GCT GTG AAG GTT GCT GTT CCT CAT | /56-FAM/TCC AGA ATC TGT TCC AGA GCG TGC |
| Skewed XCI, retinitis pigmentosa 2 gene microsatellite | /56-FAM/TGA CAT AGC GAG ACC CTG TG | GTG GTG GGT TCT CTA GCT GG |
| FLA on zebrafish, target one (exon seven) | CTG CGG GTG ACA TTG AAT C | GAC ATG CCT GGT CTA AGT GGA |
| Sanger sequencing on zebrafish, target one (exon seven) | CTT GCG GTT GTT TGG TCC TG | AGC CCA GGT GTG TCT GAT TG |
| FLA on zebrafish, target two (exon eight) | CAG GTG TCT GGT GAA GGT GAT A | AGG AGT TGC CAT TTT TGT CAC T |
| Sanger sequencing on zebrafish, target two (exon eight) pair one | TGG AAA GAA CAT GCC ATC TG | TGG TTC AGA AGC TGG AGG AG |
| Sanger sequencing on zebrafish, target two (exon eight) pair two | TGA TGG TGT CAG AAA GAA AAC A | AAA CAG CTC ATC CTG CAT TG |

**Table S3.** Overview clutches of zebrafish embryos used for quantification experiments. Genotyping of 228 zebrafish embryos display a classical Mendelian distribution, 27% wild-type (*taf1*^+/+^), 48% heterozygous (*taf1*^-/+^) and 25% homozygous (*taf1*^-/-^) embryos.

|  |  |  | |  | |  | |  |
| --- | --- | --- | --- | --- | --- | --- | --- | --- |
| **Clutch** | **Wild-type** | | **Heterozygous** | | **Homozygous** | | **Total** | **Used for quantifying** |
| 1 | 12 | | 22 | | 9 | | 43 | uu1941 eye, ear, length |
| 2 | 14 | | 21 | | 10 | | 45 | uu1941 heart beat |
| 3 | 6 | | 16 | | 7 | | 29 | uu1941 (PTU) tectum and head size |
| 4 | 7 | | 6 | | 11 | | 24 | uu1941 lethality 5dpf |
| 5 | 14 | | 20 | | 11 | | 45 | uu1931 eye, ear, length |
| 6 | 9 | | 24 | | 9 | | 42 | uu1931 (PTU) head size |
|  | 62 | | 109 | | 57 | | 228 |  |
|  | **27%** | | **48%** | | **25%** | | **100%** |  |
|  |  |  | |  | |  | |  |
| 5 | 14 | 19 | | 11 | | 44 | | uu1931 heart beat |
|  |  |  | |  | |  | |  |

**Table S4.** Raw p-values and adjusted p-values (Bonferroni correction) for boxplots displayed in Fig. 3 and Fig. S3. Wild-type (wt) = *taf1*^+/+^, mutant (mut) = *taf1*^-/-^, sibling (sib) = *taf1*^-/+^ and *taf1*^+/+^ combined. Significant p-values <0.01 in black, non-significant p-values >0.09 in grey.

|  |  |  |
| --- | --- | --- |
| **Student t-test** | **Raw p-value** | **Adjusted p-value** |
| length.194, wt vs. mut | 4,59E-04 | 2,752E-03 |
| length.194, wt vs. het | 5,34E-01 | 1,000E+00 |
| length.194, sib vs. mut | 2,66E-06 | 1,595E-05 |
| eye.194, wt vs. mut | 7,85E-07 | 4,707E-06 |
| eye.194, wt vs. het | 1,58E-01 | 9,468E-01 |
| eye.194, sib vs. mut | 1,32E-11 | 7,902E-11 |
| ear.194, wt vs. mut | 3,49E-10 | 2,094E-09 |
| ear.194, wt vs. het | 2,15E-01 | 1,000E+00 |
| ear.194, sib vs. mut | 2,20E-16 | 1,320E-15 |
| heart.beat.mean.194, wt vs. mut | 2,26E-02 | 1,353E-01 |
| heart.beat.mean.194, wt vs. het | 3,09E-01 | 1,000E+00 |
| heart.beat.mean.194, sib vs. mut | 7,55E-04 | 4,713E-03 |
| tectum.194, wt vs. mut | 5,75E-05 | 3,451E-04 |
| tectum.194, wt vs. het | 1,19E-01 | 7,164E-01 |
| tectum.194, sib vs. mut | 2,28E-08 | 1,369E-07 |
| head.size.194, wt vs. mut | 6,68E-06 | 4,007E-05 |
| head.size.194, wt vs. het | 9,91E-01 | 1,000E+00 |
| head.size.194, sib vs. mut | 6,92E-09 | 4,151E-08 |
| length.193, wt vs. mut | 3,76E-05 | 1,88E-04 |
| length.193, wt vs. het | 1,31E-01 | 6,56E-01 |
| length.193, sib vs. mut | 7,56E-06 | 3,78E-05 |
| eye.193, wt vs. mut | 2,05E-10 | 1,02E-09 |
| eye.193, wt vs. het | 9,15E-02 | 4,57E-01 |
| eye.193, sib vs. mut | 2,00E-12 | 1,00E-11 |
| ear.193, wt vs. mut | 2,05E-10 | 1,03E-09 |
| ear.193, wt vs. het | 8,62E-01 | 1,00E+00 |
| ear.193, sib vs. mut | 1,31E-13 | 6,57E-13 |
| heart.beat.mean.193, wt vs. mut | 9,09E-01 | 1,00E+00 |
| heart.beat.mean.193, wt vs. het | 8,44E-01 | 1,00E+00 |
| heart.beat.mean.193, sib vs. mut | 7,85E-01 | 1,00E+00 |
| head.size.193, wt vs. mut | 4,88E-06 | 2,44E-05 |
| head.size.193, wt vs. het | 8,69E-01 | 1,00E+00 |
| head.size.193, sib vs. mut | 2,16E-12 | 1,08E-11 |

**Table S5.** Transcriptome data. **(A)** Paired differential expression analysis using DESeq2 with counts from featureCounts, which reported 6,628 genes as differentially expressed (adjusted p-value < 0.01). **(B)** GSEA report 27 pathways to be up regulated (q-value <0.01) in *taf1*^uu1941/uu1941^ zebrafish embryos. **(C)** GSEA report 31 pathways to be down regulated (q-value <0.01) in *taf1*^uu1941/uu1941^ zebrafish embryos.

*See attached excel file S5.xlsx*

**Table S6.** PANTHER overrepresentation test (p<0.01 Bonferroni correction for multiple testing). Pathways enriched >2-fold in black.

| **PANTHER GO-Slim Biological Process** | **reference** | **observed** | | | **expected** | **fold change** | ***padj*** |
| --- | --- | --- | --- | --- | --- | --- | --- |
|  | | | | | | | |
| **Overrepresentation test for genes more than 4-fold upregulated (*n* = 258)** | | | | | | | |
| chromatin assembly (GO:0031497) | 30 | 9 | | | 0,16 | 54,84 | 1,35E-10 |
| chromatin organization (GO:0006325) | 263 | 18 | | | 1,44 | 12,51 | 4,09E-12 |
| DNA metabolic process (GO:0006259) | 347 | 10 | | | 1,9 | 5,27 | 6,18E-03 |
| organelle organization (GO:0006996) | 1342 | 21 | | | 7,34 | 2,86 | 3,48E-03 |
| Unclassified (UNCLASSIFIED) | 11889 | 52 | | | 65,04 | 0,8 | 0,00E+00 |
|  |  |  | | |  |  |  |
| **Overrepresentation test for genes more than 4-fold downregulated (*n* = 612)** | | | | | | | |
| neuromuscular synaptic transmission (GO:0007274) | 75 | 12 | | | 1,61 | 7,48 | 6,18E-05 |
| neuron-neuron synaptic transmission (GO:0007270) | 131 | 13 | | | 2,8 | 4,64 | 2,57E-03 |
| sensory perception (GO:0007600) | 230 | 17 | | | 4,92 | 3,45 | 4,64E-03 |
| G-protein coupled receptor signaling pathway (GO:0007186) | 415 | 28 | | | 8,88 | 3,15 | 6,12E-05 |
| synaptic transmission (GO:0007268) | 575 | 37 | | | 12,31 | 3,01 | 2,34E-06 |
| neurological system process (GO:0050877) | 970 | 57 | | | 20,76 | 2,75 | 5,73E-09 |
| ion transport (GO:0006811) | 491 | 28 | | | 10,51 | 2,66 | 1,34E-03 |
| system process (GO:0003008) | 1119 | 62 | | | 23,95 | 2,59 | 8,92E-09 |
| cell-cell signaling (GO:0007267) | 824 | 39 | | | 17,64 | 2,21 | 1,93E-03 |
| single-multicellular organism process (GO:0044707) | 1836 | 81 | | | 39,3 | 2,06 | 2,95E-07 |
| multicellular organismal process (GO:0032501) | 1838 | 81 | | | 39,34 | 2,06 | 3,06E-07 |
| cell surface receptor signaling pathway (GO:0007166) | 1330 | 57 | | | 28,47 | 2,00 | 3,58E-04 |
| cell communication (GO:0007154) | 3347 | 115 | | | 71,64 | 1,61 | 8,75E-05 |
| signal transduction (GO:0007165) | 2870 | 95 | | | 61,43 | 1,55 | 5,02E-03 |
| Unclassified (UNCLASSIFIED) | 11889 | 213 | | | 254,46 | 0,84 | 0,00E+00 |
| metabolic process (GO:0008152) | 7356 | 113 | | | 157,44 | 0,72 | 4,25E-03 |
| biosynthetic process (GO:0009058) | 2294 | 22 | | | 49,1 | 0,45 | 2,90E-03 |
| nucleobase-containing compound metabolic process (GO:0006139) | 3395 | 29 | | | 72,66 | 0,40 | 3,92E-07 |
| RNA metabolic process (GO:0016070) | 1970 | 3 | | | 42,16 | 0,07 | 9,64E-13 |
| regulation of transcription from RNA polymerase II promoter (GO:0006357) | 678 | 1 | | | 14,51 | 0,07 | 3,54E-03 |
| transcription from RNA polymerase II promoter (GO:0006366) | 859 | 1 | | | 18,39 | 0,05 | 7,66E-05 |
| transcription, DNA-dependent (GO:0006351) | 1422 | 1 | | | 30,44 | 0,03 | 5,71E-10 |
|  |  |  | | |  |  |  |
| **Overrepresentation test for all differentially expressed genes (*n* = 6628)** | | | | | | | |
| neuron-neuron synaptic transmission (GO:0007270) | 131 | 67 | | | 30,3 | 2,21 | 1,24E-04 |
| synaptic transmission (GO:0007268) | 575 | 225 | | | 132,99 | 1,69 | 1,75E-08 |
| system process (GO:0003008) | 1119 | 421 | | | 258,8 | 1,63 | 9,20E-15 |
| neurological system process (GO:0050877) | 970 | 357 | | | 224,34 | 1,59 | 3,12E-11 |
| single-multicellular organism process (GO:0044707) | 1836 | 637 | | | 424,63 | 1,5 | 1,10E-16 |
| multicellular organismal process (GO:0032501) | 1838 | 637 | | | 425,1 | 1,5 | 1,43E-16 |
| nervous system development (GO:0007399) | 415 | 144 | | | 95,98 | 1,5 | 9,07E-03 |
| cell adhesion (GO:0007155) | 478 | 162 | | | 110,55 | 1,47 | 9,29E-03 |
| biological adhesion (GO:0022610) | 478 | 162 | | | 110,55 | 1,47 | 9,29E-03 |
| cell-cell signaling (GO:0007267) | 824 | 277 | | | 190,58 | 1,45 | 2,39E-05 |
| system development (GO:0048731) | 530 | 177 | | | 122,58 | 1,44 | 7,56E-03 |
| cellular component morphogenesis (GO:0032989) | 576 | 189 | | | 133,22 | 1,42 | 9,93E-03 |
| cell differentiation (GO:0030154) | 665 | 214 | | | 153,8 | 1,39 | 8,39E-03 |
| developmental process (GO:0032502) | 1880 | 599 | | | 434,81 | 1,38 | 9,44E-10 |
| cellular component organization (GO:0016043) | 2171 | 634 | | | 502,11 | 1,26 | 2,59E-05 |
| organelle organization (GO:0006996) | 1342 | 392 | | | 310,38 | 1,26 | 1,00E-02 |
| cell communication (GO:0007154) | 3347 | 955 | | | 774,1 | 1,23 | 3,01E-07 |
| cellular component organization or biogenesis (GO:0071840) | 2302 | 657 | | | 532,41 | 1,23 | 2,14E-04 |
| cellular process (GO:0009987) | 9777 | 2695 | | | 2261,25 | 1,19 | 5,19E-23 |
| primary metabolic process (GO:0044238) | 5649 | 1476 | | | 1306,51 | 1,13 | 5,33E-04 |
| metabolic process (GO:0008152) | 7356 | 1886 | | | 1701,31 | 1,11 | 4,82E-04 |
| Unclassified (UNCLASSIFIED) | 11889 | 2289 | | | 2749,71 | 0,83 | 0,00E+00 |
| immune system process (GO:0002376) | 696 | 104 | | | 160,97 | 0,65 | 2,86E-03 |
| regulation of cell cycle (GO:0051726) | 544 | 60 | | | 125,82 | 0,48 | 4,91E-07 |
| negative regulation of apoptotic process (GO:0043066) | 476 | 39 | | | 110,09 | 0,35 | 1,40E-10 |
| immune response (GO:0006955) | 271 | 19 | | | 62,68 | 0,3 | 8,16E-07 |
|  |  |  | | |  |  |  |
| **Overrepresentation test for all genes (*n* = 23 919)** | | |  |  |  |  |  |
| nitrogen compound metabolic process (GO:0006807) | 3160 | 2796 | | | 2506,5 | 1,12 | 1,48E-03 |
| cellular process (GO:0009987) | 9777 | 8587 | | | 7755,07 | 1,11 | 7,51E-17 |
| primary metabolic process (GO:0044238) | 5649 | 4915 | | | 4480,76 | 1,1 | 1,44E-05 |
| Unclassified (UNCLASSIFIED) | 11889 | 8774 | | | 9430,3 | 0,93 | 0,00E+00 |
| regulation of cell cycle (GO:0051726) | 544 | 216 | | | 431,5 | 0,5 | 4,09E-17 |
| negative regulation of apoptotic process (GO:0043066) | 476 | 145 | | | 377,56 | 0,38 | 3,65E-25 |
| defense response to bacterium (GO:0042742) | 49 | 6 | | | 38,87 | 0,15 | 3,54E-05 |
| complement activation (GO:0006956) | 45 | 2 | | | 35,69 | 0,06 | 2,22E-07 |
| B cell mediated immunity (GO:0019724) | 45 | 2 | | | 35,69 | 0,06 | 2,22E-07 |
